# Development and in vitro investigation of a biodegradable mesh for the treatment of stress urinary incontinence

**DOI:** 10.1007/s00192-022-05160-2

**Published:** 2022-03-21

**Authors:** E. MacCraith, M. Joyce, R. J. F. C. do Amaral, F. J. O’Brien, N. F. Davis

**Affiliations:** 1 Tissue Engineering Research Group & AMBER Centre, Department of Anatomy & Regenerative Medicine, Royal College of Surgeons in Ireland, Dublin, Ireland; 2 Department of Urology, Blackrock Clinic, Dublin, Ireland

**Keywords:** Stress urinary incontinence, Tissue engineering, Degradable, Mesh, Sling, Polypropylene

## Abstract

**Introduction and hypothesis:**

The use of polypropylene (PP) mesh for stress urinary incontinence (SUI) surgery has declined because of safety concerns. The aim of this study is to evaluate a biodegradable polycaprolactone (PCL) mesh and a PCL composite mesh tissue engineered with human uterine fibroblasts (HUFs) for SUI surgery by comparing mechanical properties and in vitro biocompatibility to commercially available PP and porcine dermis (PD).

**Methods:**

The mechanical properties of four scaffold materials were evaluated: PCL, PCL-collagen-hyaluronic acid composite, acellular porcine dermal collagen (PD) (Pelvicol™) and polypropylene (Gynecare TVT™ Exact®). HUFs were seeded on separate scaffolds. After 7 and 14 days scaffolds were assessed for metabolic activity and cell proliferation using Alamar Blue, Live/Dead and PicoGreen assays. Soluble collagen production was evaluated using a Sircol assay.

**Results:**

PCL and the composite scaffold reached ultimate tensile strength (UTS) values closest to healthy pelvic floor tissue (PCL = 1.19 MPa; composite = 1.13 MPa; pelvic floor = 0.79 MPa; Lei et al. Int Urogynecol J Pelvic Floor Dysfunct. 18(6):603-7, 2007). Cells on PCL showed significantly greater cell viability than PP at day 7 (*p* < 0.0001). At D14 the composite scaffold showed significantly greater cell viability than PP (*p* = 0.0006). PCL was the best performing scaffold for soluble collagen production at day 14 (106.1 μg versus 13.04 μg for PP, *p* = 0.0173).

**Conclusions:**

We have designed a biodegradable PCL mesh and a composite mesh which demonstrate better biocompatibility than PP and mechanical properties closer to that of healthy pelvic floor tissue. This in vitro study provides promising evidence that these two implants should be evaluated in animal and human trials.

## Introduction

Stress urinary incontinence (SUI) is defined as the involuntary loss of urine that occurs with an increase in intra-abdominal pressure [1]. The recommended surgical treatment options include mid-urethral sling (MUS), autologous fascia sling, colposuspension and urethral bulking agents [2]. Since the 1990s, the most common surgical procedure worldwide for SUI has been the MUS with polypropylene (PP) mesh [3]. The choice of PP for SUI surgery was extrapolated from indications in abdominal wall hernia surgery. The FDA 510k process permits that new devices only have to show “substantial equivalence” with a previously approved product to be granted approval [4]. PP mesh was already approved for use in abdominal hernia surgery; therefore, many new PP mesh products for SUI surgery were introduced into clinical practice without clinical trial data. However, significant complications related to MUS surgery such as mesh erosion and chronic pain [5] have led to notable decreases in the overall treatment of SUI globally in many countries. In addition, the use of polypropylene MUS is currently paused or banned in some countries [6].

Complications due to PP are related to its non-degradable nature and poor biocompatibility. PP is 5-10 times stronger than healthy native pelvic floor tissue [7]. Polypropylene triggers an inflammatory response with an undesirable increased M1 macrophage:M2 macrophage ratio [8]. This theory is further strengthened by the findings of an M1 macrophage response in explants among 27 females undergoing mesh excision for complications 4.5–93 months after PP mesh insertion [9]. Animal models have demonstrated that PP elicits a site-specific response in the host because more erosions occur when implanted in the vagina compared to the abdominal wall [10].

Debilitating complications related to non-degradable PP, such as chronic pain and erosion, have led to the investigation of a number of synthetic degradable materials for SUI surgery such as polylactic acid (PLA), poly-DL-lactico-glycolic acid (PLGA) and polyamide (PA) [11]. These scaffolds have shown varying results in terms of biomechanics and biocompatibility during in vitro studies. Polycaprolactone (PCL) appears promising for SUI surgery because of its elasticity, miscibility with other polymers, flexibility and customisable degradation properties [12]. Another positive characteristic is that the material has been granted United States Food and Drug Administration (FDA) approval as an implantable device for other indications such as drug delivery systems and it has been utilised for bone regeneration and craniofacial repair surgery [13, 14]. PCL degrades over 2–3 years [15] and should therefore avoid complications such as chronic pain and erosion [16]. Cells isolated from uterine biopsies have shown to be a useful source for tissue engineering purposes for a number of reasons including ease of procurement, high proliferation rate, improved neo-tissue formation and anti-inflammatory effects [17]. Accordingly, in this study we used human uterine fibroblasts (HUF) as a source of donor cells. We also hypothesised that a composite scaffold of PCL reinforced with bovine collagen and hyaluronic acid may demonstrate superior mechanical properties and biocompatibility. The aim of this study is thus to evaluate the suitability of a biodegradable PCL mesh tissue-engineered with HUFs for SUI surgery by comparing mechanical properties and in vitro biocompatibility to commercially available polypropylene.

## Methods

### Overview of experimental design

Uniaxial mechanical testing of the four materials was performed. Following this, human uterine fibroblasts were isolated, cultured and seeded onto the materials. After 7 and 14 days of incubation, scaffolds were assessed for cell viability using a live/dead assay (Invitrogen, UK), metabolic activity using an Alamar Blue assay (Invitrogen, UK), DNA quantification using a Quant-iT dsDNA PicoGreen assay (Invitrogen, UK) and soluble collagen production using a Sircol collagen assay (Biocolor, UK).

### Scaffold materials

For the purpose of this study four materials were assessed. Polypropylene (Gynecare TVT ™ Exact ®) was obtained from Johnson & Johnson (New Brunswick, NJ, USA) and acellular porcine dermal collagen (PD) (Pelvicol™) was obtained from Bard (Covington, GA, USA). PD was evaluated in this experiment as it is known to have favourable in vivo biocompatibility [18]. However, PD is no longer recommended for SUI in the National Institute for Health and Care Excellence (NICE) guidelines following the PP mesh controversy [19]. Polycaprolactone (PCL) (Polysciences Inc., Warrington, PA, USA) scaffolds were 3D printed using a fused deposition modelling (FDM) printer (Allevi, Philadelphia, PA, USA ) using established techniques in our laboratory [20]. PCL and collagen hyaluronic acid composite scaffolds were fabricated using a -20 °C degree freeze drying cycle for 48 h again using established techniques in our laboratory [21].

### Mechanical assessment of scaffolds

Scaffold mechanical properties were assessed on a Zwick Roell Z005 mechanical tester (Zwick Testing Machines Ltd., Herefordeshire, UK) with a 50N load cell. All four materials were tested five times using new samples each time. Samples were cut into 1 cm × 1 cm pieces. Thickness was 0.6 mm for PCL, the composite and PP. Thickness was 0.8 mm for PD. Samples were placed vertically between two clamps and subjected to uniaxial tensile strain at an extension rate of 10 mm/min until breakage. Data were plotted on a stress-strain curve so that ultimate tensile strength (UTS) could be calculated from the plateau of the curve. Elastic modulus (EM) was calculated from the linear gradient of the curve. Yield strength (YS) was calculated as the stress under which the stress-strain curve deviates from proportionality.

### Fibroblast isolation and culture

Fibroblast isolation and culture were performed by methods previously described by Davis et al. [22]. All chemical reagents were obtained from Sigma-Aldrich® unless indicated. Human uterine fibroblasts (HUFs) (PromoCell, Heidelberg, Germany) were cultured in a humidified atmosphere of 20% oxygen, 75% nitrogen and 5% carbon dioxide in T-75 vented cap flasks (Sarstedt, Wexford, Ireland). The cell line was grown to confluence in Dulbecco’s Modified Eagle Medium (DMEM) supplemented with 10% fetal bovine serum (FBS), 1% penicillin/streptomycin solution and 0.2% primacin solution. For expansion, cell culture media were changed every 48 h until 85–90% confluency. Passage 6 HUFs were used in the present study. Each experimental sample group underwent three biological repeats (*n* = 3).

### Cell seeding

Cell seeding was performed as previously described by Davis et al. [22]. HUFs were cultured onto PP, PCL, the composite and PD. The biomaterials were cut into 1 cm^2^ squares and transferred onto 24-well tissue culture plates; HUFs were then seeded onto the surfaces of each scaffold. Specimens were seeded with 375,000 cells per cm^2^ per scaffold and cultured in 2 ml media (DMEM and FBS). Samples were cultured for 14 days and media was changed every 72 h.

### Cellular in vitro studies

After 7 and 14 days of incubation, live/dead assay for cell viability was carried out using Live/Dead Kit (Invitrogen, UK); cells were observed under a Nikon 90i microscope (Nikon, Japan).

After 7 and 14 days, the metabolic activity of the HUFs was assessed using an Alamar Blue kit (Invitrogen, UK). In short, 0.5 ml 10% Alamar solution in cell culture media was added to the wells and incubated for 1 h under standard cell culture conditions (37°C, 5% CO_2_). Media were removed from each well and fluorescence was read at 560/590 nm (excitation/emission). Acellular controls were also assessed.

After 7 and 14 days of incubation, a Quant-iT dsDNA PicoGreen assay for cellular DNA quantification was carried out using a Quant-iT Pico-Green dsDNA kit (Invitrogen, UK): media were removed from the wells and the scaffolds were placed in tubes containing 1 ml of lysis buffer [0.2 M sodium carbonate (Na_2_CO_3_) + 1% Triton X in dH_2_O]. The samples underwent three freeze-thaw cycles at -80°C before the assay was performed as per manufacturer’s instructions. The DNA concentration was determined using a standard curve provided by the manufacturer.

After 7 and 14 days of incubation, a Sircol collagen assay (Biocolor, UK) was carried out for soluble collagen production, as per manufacturer’s instructions. Absorbance was read in a microplate reader set to 555 nm.

### Statistical analysis

Statistical analysis was performed using GraphPad Prism 8 (GraphPad Software, San Diego, CA, USA). One-way analysis of variance (ANOVA) tests and Tukey’s post hoc analysis were used to determine differences between groups. *P* < 0.05 was considered statistically significant.

## Results

### Mechanical properties

**Fig. 1:**
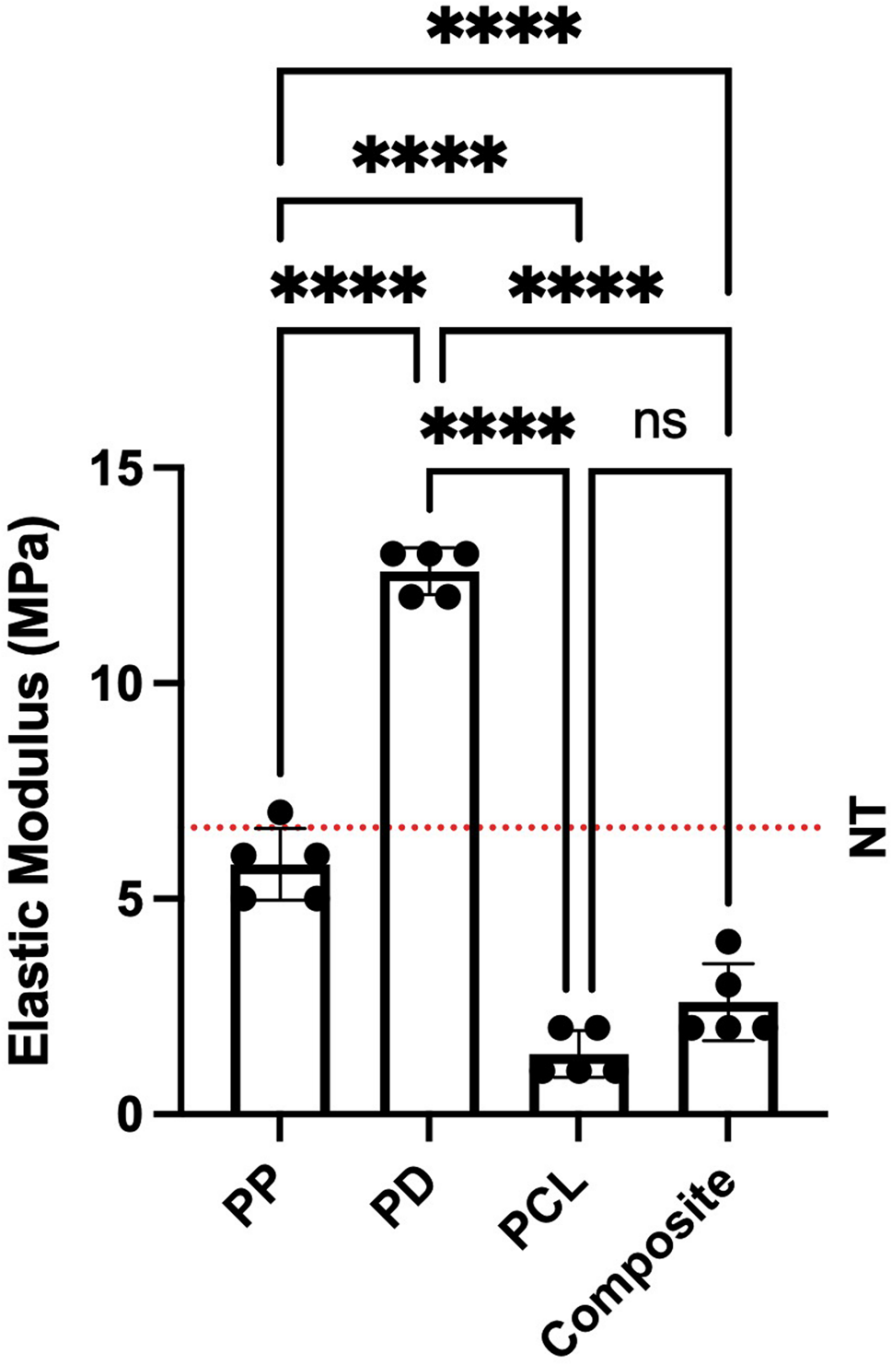
Comparison of the elastic modulus of the four candidate scaffolds demonstrating PCL and the composite are significantly less stiff than PP. Dashed line indicates values for healthy native tissue [23]. Asterisk indicates the level of significance of differences (*p < 0.05, **p < 0.01, ***p < 0.001, ****p < 0.0001). NT = native tissue, PCL = polycaprolactone, PP = polypropylene, PD = porcine dermis, ns = not significant

There was no significant difference in EM between PCL and the composite (1.4 vs. 2.6 MPa, *p* = 0.0789). PCL was significantly less stiff than PP (1.4 vs. 5.8 MPa, *p* < 0.0001) and PD (1.4 vs. 12.6 MPa, *p* < 0.0001). PP was similar to healthy pelvic floor tissue with respect to EM (5.8 versus 6.65 MPa). This is demonstrated in Fig. 1 and the dashed line indicates the previously published data for healthy pelvic floor tissue [23].

**Fig. 2:**
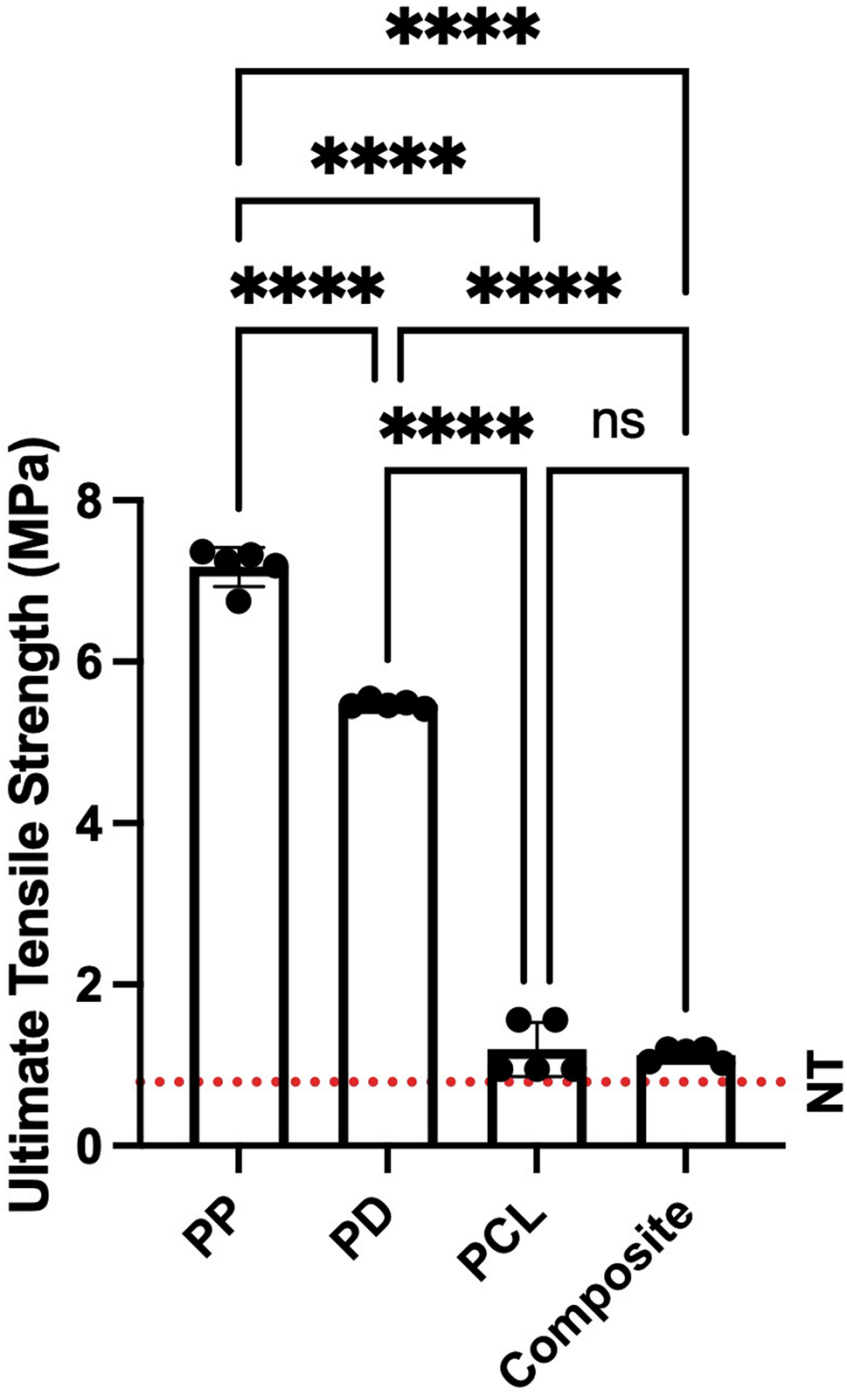
Comparison of the ultimate tensile strength of the four candidate scaffolds demonstrating that PCL and the composite are closest to healthy pelvic floor tissue. Dashed line indicates values for healthy native tissue [23]. Asterisk indicates the level of significance of differences (*p < 0.05, **p < 0.01, ***p < 0.001, ****p < 0.0001). NT = native tissue, PCL = polycaprolactone, PP = polypropylene, PD = porcine dermis, ns = not significant

For UTS both PCL and the composite scaffold were closest to values for healthy pelvic floor tissue (PCL = 1.19 MPa; composite = 1.13 MPa; pelvic floor tissue = 0.79 MPa)[23]. Again, there was no difference between PCL and the composite (1.19 vs. 1.13 MPa, *p* = 0.957). The UTS of PP was significantly greater than that of PCL (7.17 vs. 1.19 MPa, *p* < 0.0001) and greater than reported values for healthy pelvic floor tissue (7.17 vs. 0.79) [23]. These findings are illustrated in Fig. 2.

For YS there was no significant difference between PCL and composite (0.96 vs. 0.92 MPa, *p* = 0.9978). The YS of PCL was significantly less than that of PP (0.96 vs. 3.26 MPa, *p* < 0.0001) and PD (0.96 vs. 5.47 MPa, *p* < 0.0001). There are no YS data available for healthy native tissue.

### Metabolic activity

**Fig. 3:**
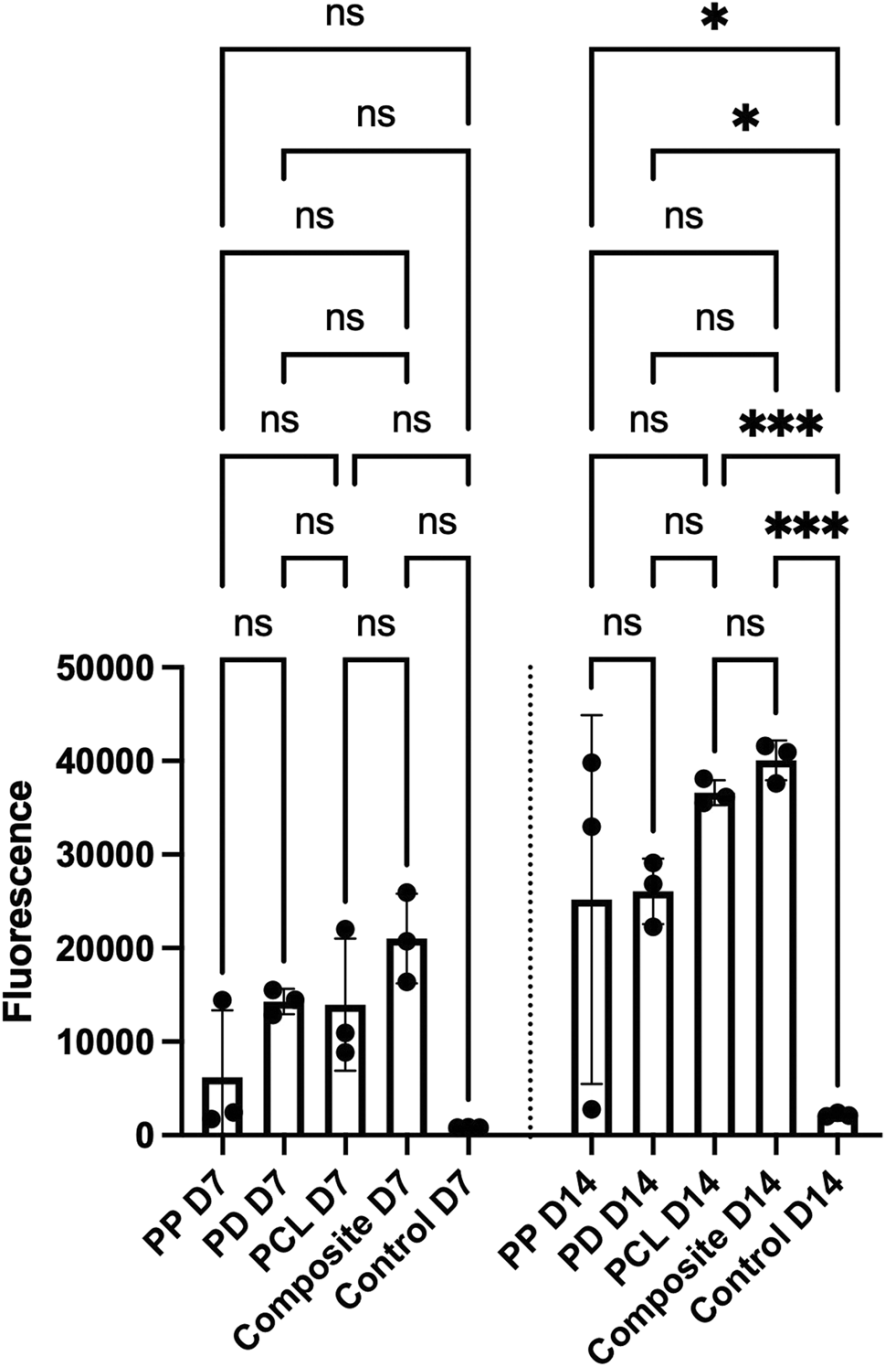
Comparison of the metabolic activity among the four scaffolds at day 7 and 14 using an Alamar Blue assay which demonstrates no significant difference between the materials. Asterisk indicates the level of significance of differences (*p < 0.05, **p < 0.01, ***p < 0.001, ****p < 0.0001). D7 = day 7, D14 = day 14, PCL = polycaprolactone, PP = polypropylene, PD = porcine dermis, ns = not significant

The metabolic activity on scaffolds was measured and on day 7 and 14 using an Alamar Blue assay, and the results are given in Fig. 3. There was no significant difference in the metabolic activity on the four scaffolds at any of the time points. The metabolic activity on all four scaffolds was significantly greater than on acellular controls at day 14. PCL was the only scaffold on which there was a significant increase in metabolic activity between day 7 and 14 (*p* = 0.03).

### Cell viability and DNA quantification

**Fig. 4:**
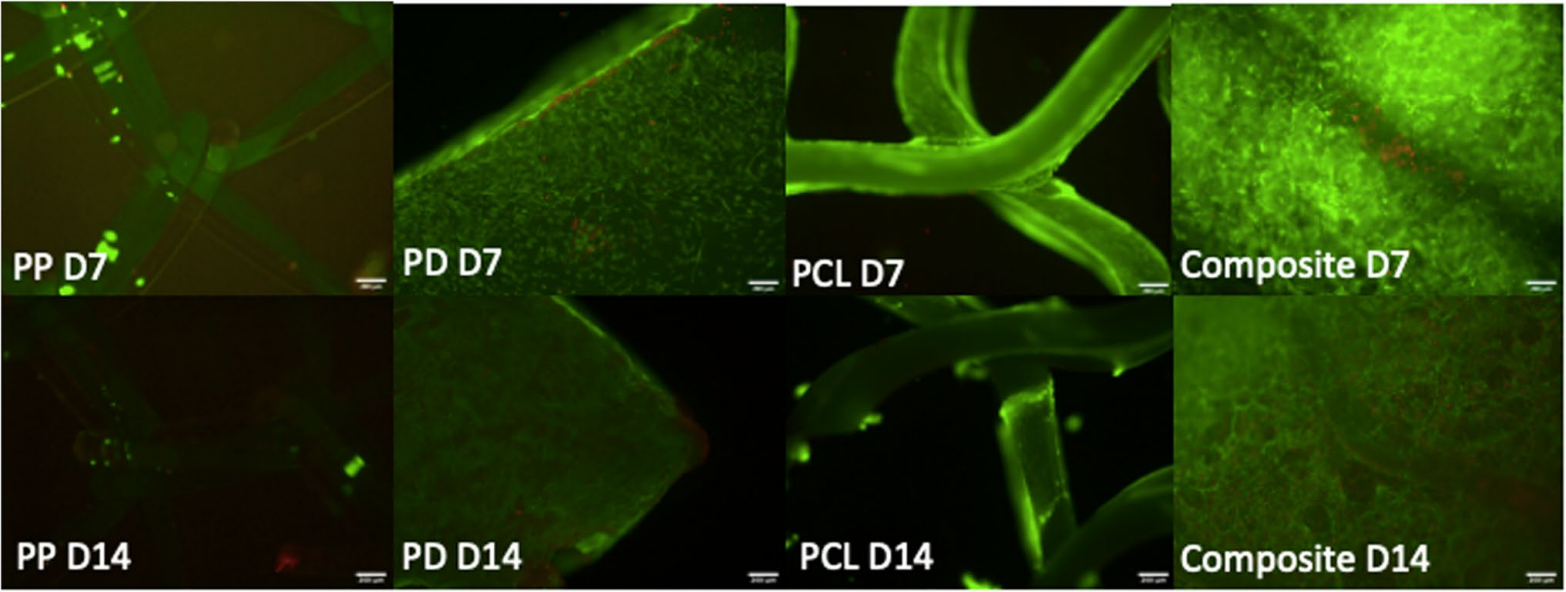
Comparison of cell viability among the four scaffolds at day 7 and 14 using a live/dead assay which demonstrates more live cells on PCL, the composite and PD compared to PP. Live cells are green and dead cells are red. Scale bar = 200 μm. D7 = day 7, D14 = day 14, PCL = polycaprolactone, PP = polypropylene, PD = porcine dermis

**Fig. 5:**
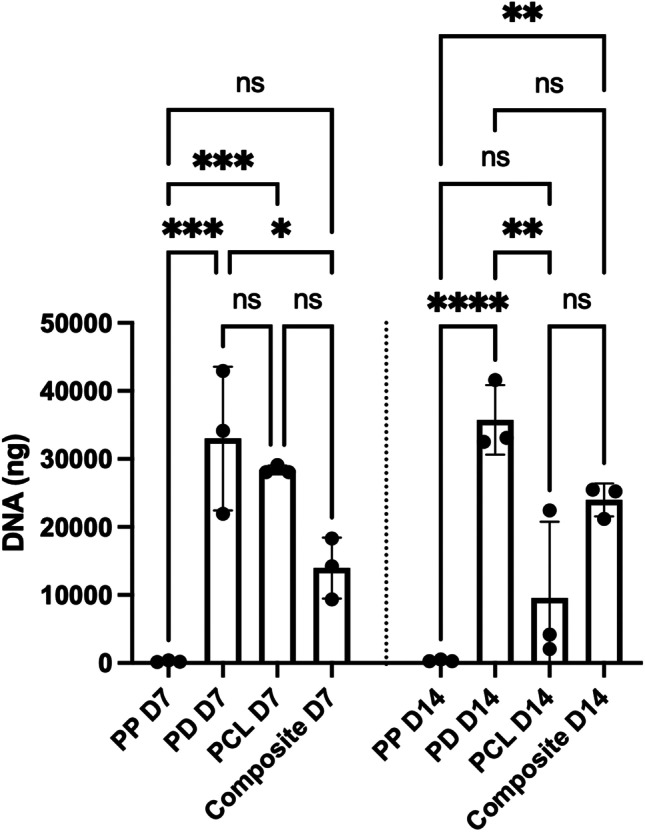
The composite scaffold demonstrated significantly greater DNA quantity compared to PP at day 14. Asterisk indicates the level of significance of differences (*p < 0.05, **p < 0.01, ***p < 0.001, ****p < 0.0001). D7 = day 7, D14 = day 14, PCL = polycaprolactone, PP = polypropylene, PD = porcine dermis, ns = not significant

Figure 4 demonstrates live/dead assay microscopy images of the four scaffolds at day 7 and 14. PCL demonstrates more live cells at day 14 than PP. A PicoGreen assay was performed to evaluate DNA quantity at day 7 and day 14 (Fig. 5). At D14 the composite scaffold showed significantly greater cell viability than PP (*p* = 0.0006); no difference was observed between PCL and PP at this time point (*p* = 0.555). PCL showed significantly greater cell viability than PP at day 7 (*p* < 0.0001). PD showed significantly greater cell viability than PP at day 14 (*p* < 0.0001).

### Soluble collagen production

**Fig. 6:**
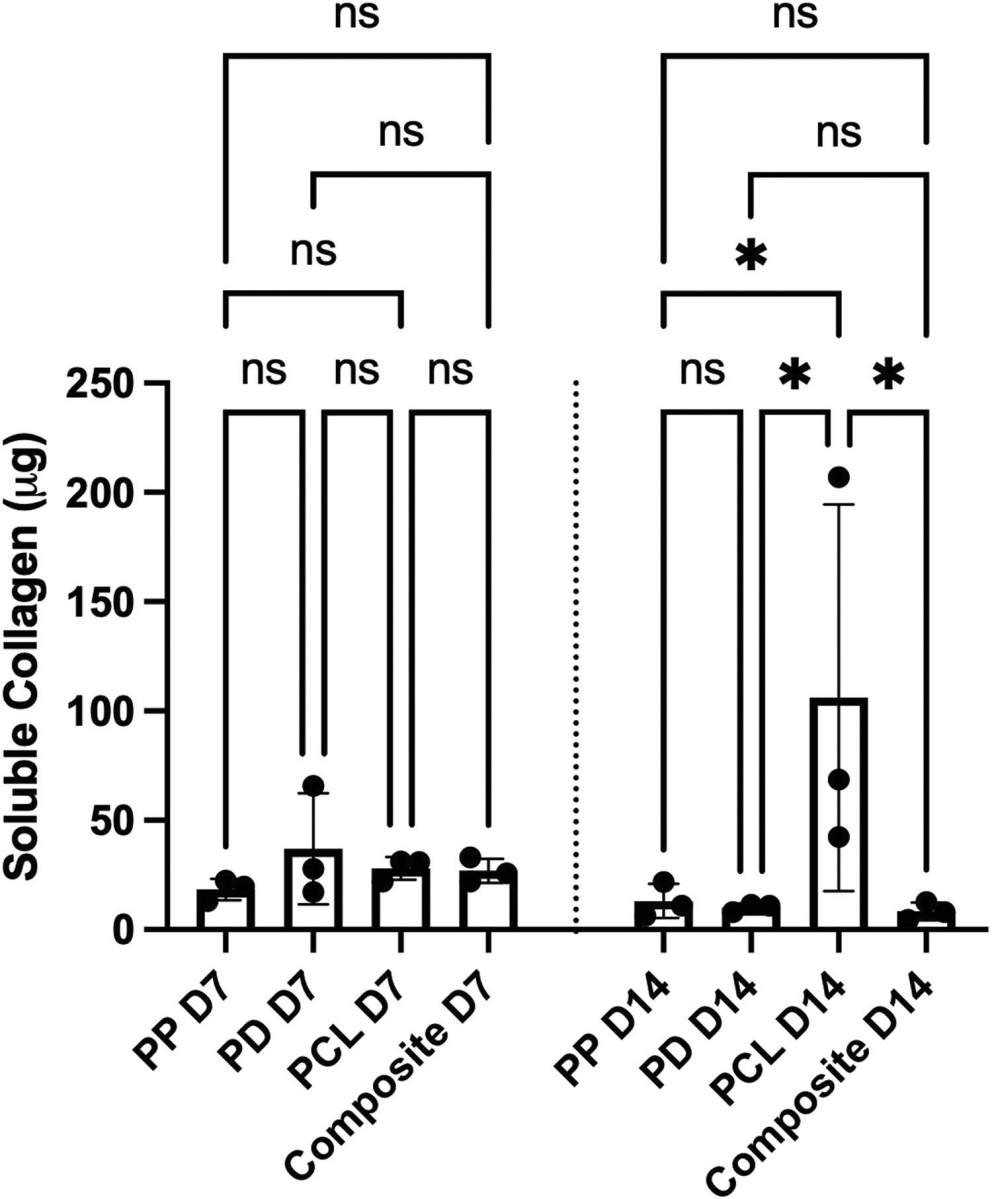
Cells on PCL demonstrated significantly greater collagen production compared to all other materials at day 14. Asterisk indicates the level of significance of differences (*p < 0.05, **p < 0.01, ***p < 0.001, ****p < 0.0001). D7 = day 7, D14 = day 14, PCL = polycaprolactone, PP = polypropylene, PD = porcine dermis, ns = not significant

A Sircol collagen assay was performed at day 7 and 14 to assess cellular soluble collagen production. The results are demonstrated in Fig. 6. The best performing scaffold was PCL. At the day 14 final time point PCL showed significantly greater soluble collagen production than PP (*p* = 0.017), PD (*p* = 0.013) and the composite (*p* = 0.011).

## Discussion

The aim of this study was to evaluate the suitability of a biodegradable PCL mesh for SUI surgery by comparing mechanical properties and in vitro biocompatibility to commercially available polypropylene and porcine dermis. We designed a biodegradable PCL mesh and a PCL and collagen hyaluronic acid composite mesh, both of which demonstrate better biocompatibility than PP. The PCL and composite mesh also demonstrate mechanical properties similar to healthy pelvic floor tissue. We demonstrated that PCL and the composite mesh have an equivalent biocompatibility to PD, which is important, as this implant has an excellent safety profile in humans [18]. The PCL mesh also outperformed all other candidate scaffolds in terms of collagen production. This will be important after 2 years when the material degrades.

In this study PCL demonstrated superior biocompatibility to PP in a number of ways. PCL was the only scaffold on which there was a significant increase in metabolic activity; PCL demonstrated more live cells than PP at day 14, significantly greater cell viability than PP at day 7 and significantly greater soluble collagen production at day 14. Studies comparing PCL to PP have demonstrated conflicting results. Hympanova et al. evaluated PCL modified with ureidopyrimidinone motifs (UPy-PCL) in animal models [24]. The authors implanted UPy-PCL and a control group of PP mesh into abdominal wall defects in rats. Both meshes had identical erosion rates (2/12 cases). The UPy-PCL explants exhibited an undesirable M1-dominated host response. Our findings support recent findings on the promising biocompatibility and mechanical suitability of PCL-based implants. Paul et al. evaluated PCL encapsulated in an aloe vera-sodium alginate hydrogel [25]. The authors hypothesized that this would promote tissue integration while controlling the foreign body response. The mesh was seeded with endometrial mesenchymal stem cells (MSCs) and then implanted into subcutaneous tissue in the abdominal wall of mice. There was a favourable M2 inflammatory response with complete integration of the mesh and deposition of ECM.

Mangera et al. compared PLA with cadaveric dermis, porcine dermis, polypropylene and porcine small intestinal submucosa (SIS) for SUI surgery [26]. The authors seeded oral mucosal fibroblasts onto scaffolds. Similar to the present study, PLA was the optimal material in terms of cell attachment, cell proliferation and ECM production with mechanical properties that were most similar to native tissue. Both studies evaluated candidate scaffolds for 14 days. However, we selected a source of fibroblasts (HUFs) (PromoCell, Heidelberg, Germany), which we felt were more relevant to the implant destination. In addition, these cells have a number of advantages of other fibroblast sources, including ease of procurement, high proliferation rate, improved neo-tissue formation and anti-inflammatory effects [17]. Both PCL and PLA appear to be promising candidate materials for SUI and are already approved for use in humans for other indications.

Hung et al. seeded adipose-derived stem cells onto PLGA scaffolds and then implanted the mesh into mice under the back skin [27]. At 4, 8 and 12 weeks after transplantation, tissue samples were harvested for histological analysis. The mesh had not fully degraded at 12 weeks. There was abundant collagen deposition and the neo-tissue formed was a well-organised lamellar structure which mimicked normal fascia. Unlike our study, analysis of mechanical properties of materials was not performed and there was no control group of PP mesh. Ulrich et al. implanted a gelatin-coated knitted polyamide (PA) mesh seeded with endometrial MSCs into the subcutaneous tissues on the back of rats [17]. They compared the seeded PA mesh to an unmodified PA mesh. The seeded explants were associated with greater neovascularisation, reduced inflammatory response, more organised collagen deposition and decreased stiffness compared to the unmodified PA mesh. Unlike our study, no control group of PP or PD material was included.

Materials for SUI surgery should be elastic to cope with increased abdominal pressure with coughing and sneezing and should be stronger at higher strain like fascia [28]. PP is stronger and stiffer than healthy pelvic floor tissues and this is likely one of the factors contributing to complications such as erosion and pain [7]. In our study the PCL and composite mesh had significantly lower EM compared to PP. This may be a positive finding in terms potential for mesh erosion. It also may be a negative finding in terms of likelihood of mesh failure and SUI recurrence.

Our study is limited by the fact that the Sircol assay only detects soluble collagen. This would not accurately identify the insoluble collagen contained in both the PD and the composite scaffolds. Results of the mechanical assessment of scaffolds in this study must be interpreted with caution as the materials are not solid materials. The authors acknowledge that since, in certain jurisdictions, autologous fascial sling is the gold standard operation for SUI since the ban on PP that we would have included human fascia as a control group in the current study. Future studies should characterise the type of inflammatory response generated by the PCL implant as this will be important in determining the long-term sub-urethral mechanical support that exists following degradation of PCL. These investigations would ideally be conducted in animal models with a follow-up duration long enough to quantify mechanical and histological properties of explant tissues post-PCL degradation.

## Conclusion

We have designed a biodegradable PCL mesh and a PCL and collagen hyaluronic acid composite mesh, which demonstrate superior biocompatibility than PP and mechanical properties closer to that of healthy pelvic floor tissue, and demonstrates cellular support which is equivalent to PD, an implant with an excellent safety profile in humans. The PCL material also outperformed all other candidate scaffolds in terms of collagen production. This in vitro study provides promising evidence that these two implants should be evaluated in animal and human trials with a view to integrating it into the care pathway for female patients with SUI in future.

## Abbreviations

SUI: stress urinary incontinence
MUS: mid-urethral sling
PP: polypropylene
UBA: urethral bulking agent
PLA: polylactic acid
PLGA: poly-DL-lactico-glycolic acid
PA: polyamide
PCL: polycaprolactone
HUFs: human uterine fibroblasts
UTS: ultimate tensile strength
EM: elastic modulus
YS: yield strength
FCM: fibroblast culture medium
FBS: fetal bovine serum
DMEM: Dulbecco’s Modified Eagle Medium
MSC: mesenchymal stem cells
